# Type I interferon mitigates ZIKV-induced neuroinflammation and cell death in the central nervous system

**DOI:** 10.1016/j.virusres.2026.199814

**Published:** 2026-10-04

**Authors:** Luciana Benevides, Herculano da Silva, Franciele Pioto, Thais da Silva Rigoni, Mariela Pires Cabral Piccin, Juliano de Paula Souza, Eurico Arruda, Vanessa Carregaro, João S. Silva

**Affiliations:** a Fiocruz-Bi-Institutional Translational Medicine Plataform, Ribeirão Preto, São Paulo, Brazil; b Department of Biochemistry and Immunology, Ribeirão Preto Medical School University of São Paulo, Ribeirão Preto, São Paulo, Brazil; c Department of Cell Biology and Virology Research Center, Ribeirão Preto Medical School University of São Paulo, Ribeirão Preto, São Paulo, Brazil

**Keywords:** Zika virus, IFN-I signaling, Neuroinflammation, Cell death

## Abstract

Zika virus (ZIKV) infection can cause severe neurological complications, particularly when antiviral immune responses are impaired. Here, we investigated how type I interferon (IFN-I) signaling influences the cellular inflammatory response and immunopathology in the central nervous system (CNS) during ZIKV infection. ZIKV infection induced early IFN-α and IFN-β expression in the CNS of wild-type (WT) mice. In contrast, mice lacking the IFN-I receptor (IFNAR^−/−^) exhibited higher CNS viral loads, severe clinical symptoms, weight loss, increased mortality, and histopathological alterations characterized by extensive leukocyte accumulation and ZIKV antigen detection. Flow cytometry and UMAP analysis revealed that IFN-I signaling restrains leukocyte accumulation in the CNS. IFNAR^−/−^mice showed increased accumulation of myeloid and lymphoid cells, including inflammatory monocytes, monocyte-derived dendritic cells, and activated CD4^+^ and CD8^+^ T cells, whereas WT mice displayed limited leukocyte accumulation. Loss of IFN-I signaling was associated with elevated levels of pro-inflammatory cytokines (IL-6, IL-1β, TNF, and IFN-γ), chemokines (CCL2, CCL4, and CCL5), and inducible nitric oxide synthase (iNOS). Notably, CNS-resident microglia from IFNAR^−/−^ mice were highly susceptible to cell death, including among ZIKV antigen-negative cells, suggesting that inflammatory signals generated during infection may contribute to injury in uninfected microglia. In contrast, myeloid cells were predominantly ZIKV antigen-positive but remained largely viable. CD45^−^ non-leukocyte cells constituted the major ZIKV antigen-positive population in the CNS and displayed resistance to cell death, indicating that this compartment may represent an important cellular population associated with ZIKV antigen detection during acute infection. Together, these findings demonstrate that IFN-I signaling is essential for controlling ZIKV replication, restraining neuroinflammation, and limiting CNS immunopathology. Its absence leads to increased viral burden, dysregulated immune cell accumulation, and severe neurological disease.

## Highlights

• IFNAR deficiency increases viral burden and mortality after ZIKV infection.
• Type I interferon signaling limits neuroinflammation in the CNS.
• Loss of IFN-I responses enhances neuronal damage and cell death.
• IFN-I protects the central nervous system against severe ZIKV pathology.

## 1 Introduction

Zika virus (ZIKV) is a single-stranded RNA virus belonging to the family *Flaviviridae*. Most ZIKV infections begin with the bite of a virus-infected *Aedes* mosquito (Dahiya et al., 2023). ZIKV has garnered significant attention in recent years due to its association with severe birth defects, such as microcephaly in newborns, and Guillain-Barré syndrome in adults (Guo et al., 2022). Large-scale outbreaks have been reported worldwide, leading to the declaration of ZIKV infection as a Public Health Emergency of International Concern.

Although many individuals infected with ZIKV experience mild or no symptoms, the virus can cause serious complications, particularly in pregnant women and their unborn babies. ZIKV infection during pregnancy has been strongly associated with congenital Zika syndrome, which comprises a spectrum of abnormalities, including severe brain malformations, ocular defects, and other neurological impairments in newborns (Giraldo et al., 2023).

The host innate immune response represents the first line of defense against ZIKV infection. Among innate immune pathways, type I interferon (IFN-I) signaling plays a pivotal role in restricting viral replication and spread. Upon recognition of viral RNA by pattern recognition receptors, infected host cells initiate cellular responses aimed at limiting viral replication and dissemination. A key component of this response is the production of IFN-I, such as interferon-alpha (IFN-α) and interferon-beta (IFN-β), which induce the expression of interferon-stimulated genes that establish an antiviral state in both infected and neighboring cells (Hu et al., 2023; Rojas et al., 2021).

Despite the potency of IFN-I-mediated antiviral responses, ZIKV, like other flaviviruses, has evolved strategies to evade or antagonize IFN-I signaling, thereby enhancing viral replication and persistence within the host. Viral proteins have been shown to interfere with IFN-I production and downstream pathways, facilitating viral dissemination and persistence in host tissues, including immune-privileged sites such as the central nervous system (CNS) (Rojas et al., 2021). This immune evasion is thought to contribute to ZIKV neuropathogenesis. Consequently, a deeper understanding of the interaction between ZIKV and IFN-I pathways is critical for the development of effective therapeutic strategies and may provide broader insights into the mechanisms underlying virus-induced neurological damage.

Animal models have been instrumental in elucidating the role of IFN-I signaling in ZIKV infection. Lazear et al. demonstrated that Ifnar1^−/−^ mice develop severe neurological disease and sustain high viral loads in the brain and spinal cord following ZIKV infection, whereas immunocompetent wild-type mice show limited disease (Lazear et al., 2016). Similarly, Rossi et al. showed that ZIKV caused severe disease and mortality in IFNAR-deficient A129 mice but not in age-matched immunocompetent mice, further establishing the critical role of IFN-I signaling in restricting ZIKV pathogenesis (Rossi et al., 2016). These models provide valuable insights into the mechanisms by which IFN-I responses regulate viral tropism, neuroinflammation, and tissue damage within the CNS.

Although the antiviral role of type I interferon (IFN-I) signaling in controlling ZIKV replication and disease severity is well established, less is understood about how IFN-I signaling influences the cellular inflammatory response within the central nervous system (CNS) during infection (Lazear et al., 2016; Jurado et al., 2018). In particular, the extent to which IFN-I signaling influences the recruitment and accumulation of distinct myeloid and lymphoid populations in the CNS remains incompletely characterized (Hayashida et al., 2019). Understanding this relationship is important because excessive or dysregulated neuroinflammation may contribute to CNS pathology in addition to the effects of viral replication. In this context, we investigated the temporal IFN-I response and the relationship between viral burden, CNS leukocyte infiltration, and disease progression following ZIKV infection in wild-type and IFNAR-deficient mice. Our study was designed not only to confirm the established antiviral function of IFN-I but also to characterize its contribution to the cellular neuroinflammatory response during ZIKV infection.

Our findings demonstrate that IFN-I signaling plays a critical role in controlling ZIKV infection in the CNS and in regulating the magnitude and composition of the inflammatory response. Mice lacking the IFN-I receptor exhibited increased viral loads, exacerbated neuroinflammation, excessive leukocyte infiltration, and increased cell death compared with wild-type controls. Together, these findings provide an integrated characterization of the relationship between IFN-I signaling, viral burden, CNS inflammation, and disease progression during ZIKV infection.

## 2 Materials and methods

### 2.1 Animals

Female C57BL/6 (WT) and IFNAR^−/−^ mice (deficient in the type I interferon receptor) were bred and maintained in a specific pathogen-free facility at the University of São Paulo, Brazil. IFNAR^−/−^ mice on a C57BL/6 genetic background and wild-type (WT) C57BL/6 mice were used in this study. Female mice aged 3–4 weeks were used for all experiments. All experimental procedures were conducted in accordance with international guidelines for the use of laboratory animals and approved by the local Ethics Committee at the University of São Paulo, Brazil (protocol number 152/2017).

### 2.2 Virus stocks and mouse infection

ZIKV strain BEH819966, originally isolated from a patient in the city of Belém, Pará state, Brazil, was obtained from Instituto Evandro Chagas. Virus stocks were propagated in Vero cells cultured in Dulbecco’s Modified Eagle Medium (DMEM) supplemented with 2 % heat-inactivated fetal bovine serum (Gibco). Viral titers were determined by plate assay in Vero cells overlaid with 1 % carboxymethyl cellulose and incubated for six days. Mice were inoculated intraperitoneally with 1 × 10^5^ plaque-forming units (PFU) of ZIKV diluted in phosphate-buffered (PBS). Animals were monitored three times daily for clinical signs of disease, and clinical score was assigned at each observation (0 normal; 1 lethargy, 2 pronounced lethargy, 3 reduced surveillance, 4 ruffled fur; 5 hunched and labored breathing, and 6 immobile), as previously described (Chan et al., 2016). Body weight and survival were monitored daily throughout the observation period. At 24, 48, and 72 h post-infection (hpi) and 5 days post-infection (dpi), CNS samples were collected for analysis of viral load by qPCR, leukocyte populations by flow cytometry, and cytokine and chemokine expression by qPCR and ELISA.

### 2.3 Quantification of viral load by qPCR

Total viral RNA was extracted from CNS tissues using the QIAamp Viral RNA Mini Kit (Qiagen) according to the manufacturer's instructions. ZIKV RNA was quantified by one-step real-time RT-PCR using the QuantiNova Probe RT-PCR Kit (Qiagen) and the StepOnePlus Real-Time PCR System (Applied Biosystems, USA). Each 20 µL reaction contained 2.5 µL RNA, 10 µL 2x Probe RT-PCR Master mix, 0.5 µL QN Probe RT-mix, 0.5 µL forward primer (5’ CCGCTGCCCAACACAAU 3’), 0.5 µL reserve primer (5’ CCACTAACGTTCTTTTGCAGACAT3’), 0.5 µL probe (FAM-labeled), and nuclease free water. Thermal cycling conditions consisted of reverse transcription at 45°C for 10 min, initial denaturation at 95°C for 5 min, and 45 cycles - 95°C for 5 s, 60°C for 30 s. Standard curves were generated using 10-fold serial dilutions ZIKV RNA concentrations.

### 2.4 Isolation of leukocytes and flow cytometry analysis

For the purposes of this study, CNS refers specifically to the whole brain, as spinal cord tissue was not collected or analyzed. To assess the inflammatory infiltrate in the CNS, whole brains were collected after intracardiac perfusion with PBS and processed as previously described (Lindsley and Rodriguez, 1989; Sedgwick et al., 1991), with minor modifications. Briefly, mice were deeply anesthetized and perfused intracardially with 50 ml PBS. Whole brains were collected, minced in Hank’s balanced salt solution, and passed through a 70 µm cell strainer to obtain a single-cell suspension. Cells were washed separated by centrifugation over a 30–70 % Percoll gradient (GE Healthcare) at 1250 × g for 45 min. Mononuclear cells collected from the interphase were washed in PBS and counted by trypan blue exclusion. For surface staining, 0.5–2 × 10^6^ cells were incubated with Fc block (anti-CD16/CD32) for 20 min at 4°C. Cells were then stained following fluorochrome-conjugated antibodies (from BD Biosciences and Biolegend): CD45 (16A), CD11b (M1/70), CD11c (N418), MHCII (M5/114.15.2), Ly6C (HK1.4), CD3 (145–2C11), CD4 (RM4-5), CD8 (53–6.7), CD44 (IM7) for 30 min at 4°C in the dark.

Intracellular staining for ZIKV was performed using a primary antibody against ZIKV proteins (1:100, GeneTex) following cell fixation with 4 % paraformaldehyde (PFA) and permeabilization with 0.5 % saponin. After washing, cells were incubated with Alexa Fluor 488-conjugated anti-rabbit secondary antibody (1:500, Abcam) for 30 min at 4°C in the dark. Cell viability and death were assessed with Annexin V and 7-Aminoactinomycin D (7-AAD) according to the manufacturer’s instructions. Annexin V was used to identify early apoptotic cells, while 7-AAD staining allowed for the detection of late apoptotic and necrotic cells.

The high-dimensional analyses corresponded to two distinct analytical approaches. For the characterization of the CNS inflammatory infiltrate, dimensionality reduction and unsupervised clustering were performed using selected leukocyte lineage and phenotypic markers, including CD45, CD11b, CD11c, MHC-II, Ly6C, CD3, CD4, CD8, and CD44. The resulting clusters were annotated based on their characteristic marker-expression profiles and established immunophenotypic definitions. Cluster identities were subsequently compared with conventional manual gating to assess consistency between the unsupervised and manually defined populations.

For the second analysis, ZIKV antigen expression and cell viability were evaluated to characterize the infection and viability status of the identified cellular populations. Annexin V and 7-AAD were used to assess apoptosis and necrosis, respectively. ZIKV antigen expression was subsequently used to determine the infection status of the identified populations.

Samples were acquired on a FACSCanto II or FACSymphony A1 flow cytometer and analyzed using FlowJo software. Dimensionality reduction and clustering analyses were performed using FlowJo plugins, including UMAP, X-Shift, FlowSOM, and Cluster Explorer. UMAP was used for dimensionality reduction and visualization of the cellular landscape, whereas X-Shift and FlowSOM were used for unsupervised clustering. Cluster Explorer was used to explore and visualize the resulting clusters and their marker-expression profiles.

### 2.5 Histological and immunostaining analysis

For all histological and immunostaining analyses, CNS refers to whole-brain tissue, as spinal cord tissue was not collected or analyzed.

For histological analysis, CNS tissues were fixed in 10 % buffered formalin, paraffin-embedded, sectioned at 5-µm, and stained with hematoxylin and eosin (H&E).

For immunohistochemistry (IHC), CNS tissues were embedded in OCT compound (Sakura Finetek), snap-frozen in liquid nitrogen, and stored at −80°C. Frozen sections (5 μm) were fixed with acetone and incubated with 3 % hydrogen peroxide to block endogenous peroxidase. Sections were incubated overnight at 4°C with anti-mouse cleaved caspase-3 antibody (1:50; Cell signaling). Detection was performed using MACH 1 Universal HRP-Polymer (Biocare Medical, USA), followed by DAB substrate and counterstaining with Mayer’s hematoxylin.

For immunofluorescence (IF), frozen CNS sections (5 µm) were fixed with cold acetone, washed with PBS, and blocked with 1 % bovine serum albumin (BSA) in PBS for 30 min at room temperature. Sections were incubated overnight at 4°C with a primary antibody against ZIKV antigen (1:100, GeneTex) diluted in PBS with 0.5 % saponin. After washing, sections were incubated with Alexa Fluor 488-conjugated anti-rabbit antibody (1:500; Abcam) for 1 hour at room temperature in the dark. Nuclei were counterstained with DAPI (Prolong), and images were acquired using a fluorescence microscope.

### 2.6 Gene expression analysis by quantitative real-time PCR (qPCR)

Total RNA was extracted from CNS tissues using TRIzol reagent (Invitrogen) followed by purification with the SV Total RNA Isolation System (Promega). cDNA synthesis was performed using the High-Capacity cDNA Reverse Transcription Kit (Applied Biosystems). Quantitative real-time PCR was performed using a GoTaq qPCR Master Mix (Promega) on a StepOnePlus Real-Time PCR System. Primer sequences are listed in Supplementary Table 1. Cycling conditions were as follows: 50°C for 2 min; 95°C for 2 min; and 40 cycles of 95°C for 15 s, 58°C for 30 s, 72°C for 30 s. Gene expression levels were normalized to the housekeeping gene *Hprt* (*hypoxanthine phosphoribosyltransferase*), and relative expression calculated using the 2^−ΔCt method.

### 2.7 Cytokine and chemokine detection

CNS from ZIKV-infected mice were harvested, weighed, homogenized in lysis buffer containing protease inhibitors (Complete, Roche), and centrifuged. Cytokines (IL-6, IL-1β, TNF, and IFN-γ) and chemokines (CCL2, CCL4 and CCL5) levels were quantified in tissue supernatants by ELISA according to the manufacturer’s instructions (BD Biosbience, Biolegend and R&D Systems). Results were expressed as picograms per milliliter (pg/mL).

### 2.8 Statistical analysis

Statistical analyses were performed using one-way ANOVA followed by Tukey’s multiple comparisons test for comparisons among more than two groups, and an unpaired Student’s *t*-test for comparisons between two groups. Survival curves were analyzed using the Kaplan–Meier method and compared using the Mantel–Cox log-rank test. All statistical analyses were performed using GraphPad Prism version 5.0. Differences were considered statistically significant at *p* < 0.05.

## 3 Results

### 3.1 IFN-I signaling controls ZIKV pathogenesis

To validate our model of ZIKV-induced disease in mice, we first determined the expression of type I interferons in the CNS following ZIKV infection. Both IFN-α and IFN-β expression levels were significantly increased in CNS tissues of ZIKV-infected mice at 48- and 72- hours post-infection (hpi) (Fig. 1A). To determine the role of IFN-I signaling in disease pathogenesis, and to further validate our model, wild-type (WT) and IFNAR^−/−^ were infected with ZIKV. Unlike WT mice, which restricted viral replication in CNS and exhibited no significant weight loss or clinical symptoms, with 100 % of survival, ZIKV-infected IFNAR^−/−^ mice exhibited dramatically increased susceptibility to the infection, with higher viral loads in the CNS (Fig. 1B), pronounced weight loss (Fig. 1C), increased clinical disease scores (Fig. 1D), and complete mortality by 12 days post-infection (dpi) (Fig. 1E). Histological analysis of CNS tissues at 5 dpi revealed pronounced leukocyte infiltration in IFNAR^−/−^ mice compared to WT controls (Fig. 1F). Furthermore, ZIKV antigen was clearly detected in the CNS of IFNAR^−/−^ mice, but was absent in WT (Fig. 1G). Together, these data demonstrate that IFN-I signaling plays a critical role in restricting ZIKV replication and limiting disease severity and CNS pathology.

**Fig. 1:**
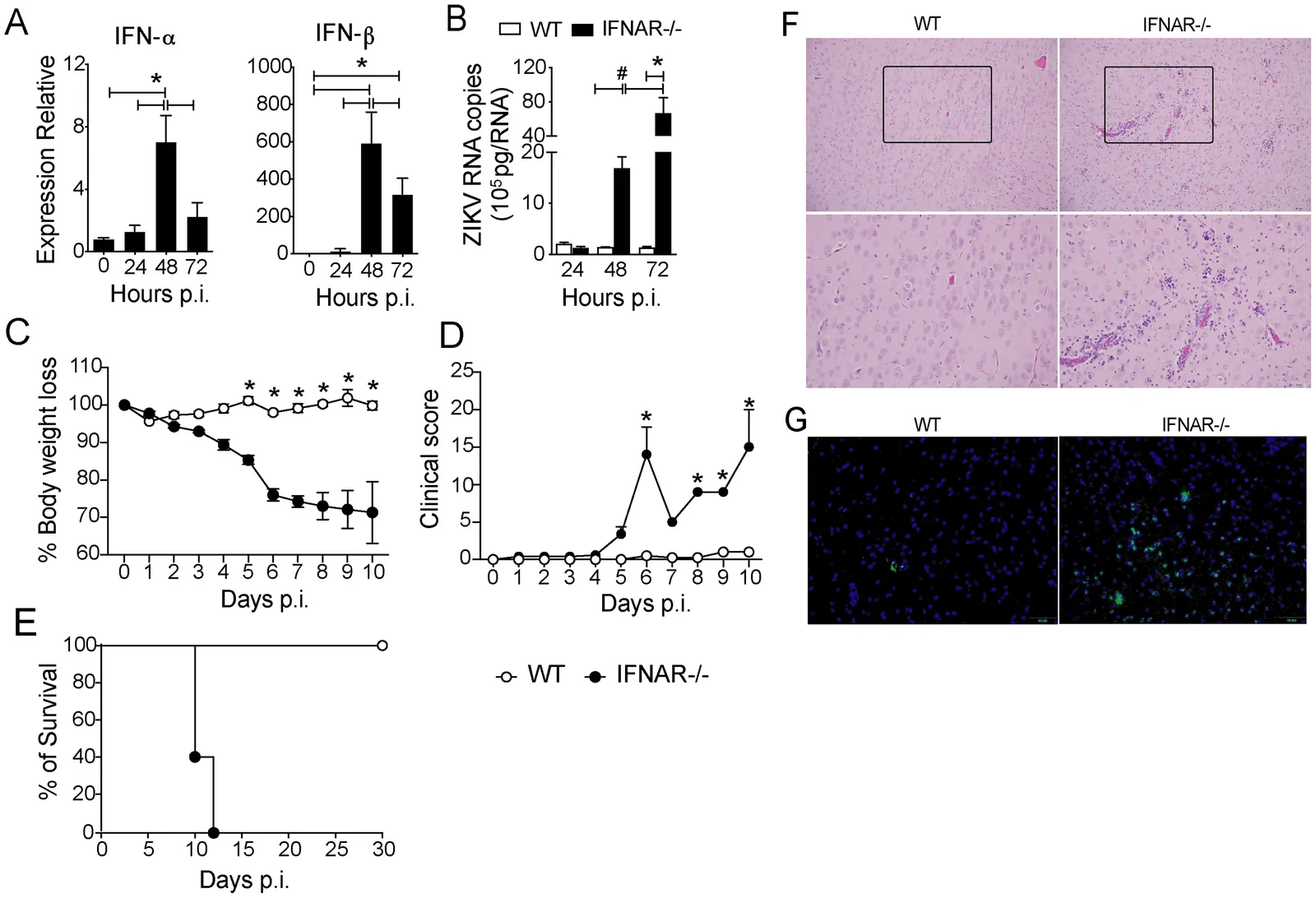
**ZIKV induces severe neuroinflammation and death in IFNAR**^−^**^/^**^−^**mice**. WT mice were infected with ZIKV, and the relative mRNA expression of IFN-α and IFN-β in central nervous system (CNS) tissue was determined at 24, 48, and 72 hours post-infection (hpi) (A). (B–E) WT (open circles) and IFNAR^−/−^ mice (closed circles) were intraperitoneally infected with 1 × 10^5^ PFU of ZIKV, and viral load in the CNS (B), body weight (C), clinical score (D), and survival rate (E) were monitored over the course of infection. Clinical scores were assigned three times daily (0 = normal; 1 = lethargy; 2 = pronounced lethargy; 3 = reduced surveillance; 4 = ruffled fur; 5 = hunched posture and labored breathing; 6 = immobile). (F) Representative images of H&E-stained CNS sections and (G) ZIKV immunofluorescence staining in WT and IFNAR−/− mice at 5 days post-infection (dpi) are shown. Scale bars = 50 μm. Data represent mean ± SEM from two independent experiments, with five mice per group. *P < 0.05.

### 3.2 Deficiency of IFN-I signaling is associated with increased leukocyte accumulation in the CNS during ZIKV infection

With our ZIKV mouse infection model established, we sought to investigate the immunopathological mechanisms underlying the observed brain lesions. We first examined how IFN-I signaling modulates leukocyte recruitment by analyzing CNS-infiltrating immune cell populations in WT and IFNAR^−/−^ mice at 5 dpi with ZIKV. The frequency of CD45^lo^CD11b^+^ microglia was comparable between WT (9.29 ± 2,08 %) and IFNAR^−/−^ (19.77 ± 3.20 %) mice (p = 0.0519). Similarly, absolute number of microglia was comparable between WT (10.31 ± 2.42 × 10^2^) and IFNAR^−/−^ (108.3 ± 36.11 × 10^2^) mice (p = 0.0537) (Fig. 2A and B). In contrast, the frequency of CD45^hi^CD11b^+^ myeloid cells (MCs) was significantly higher in IFNAR^−/−^ (3.90 ± 0.79 %) than in WT (0.57 ± 0.18 %) mice (p = 0.0147). Their absolute number was also significantly increased in IFNAR^−/−^ (21.27 ± 6.88 × 10^2^) compared with WT (0.61 ± 0.17 × 10^2^) mice (p = 0.0400) (Fig. 2A and C). Similarly, the frequency of CD45^hi^CD11b^−^ lymphoid cells (LCs) was significantly higher in IFNAR^−/−^ (13.25 ± 1.53 %) than in WT (0.87 ± 0.26 %) mice (p = 0.0013), as was their absolute number (IFNAR^−/−^: 66.27 ± 2.93 × 10^2^ vs. WT: 0.93 ± 0.25 × 10^2^, p < 0.0001) (Fig. 2A and D). These findings indicate increased leukocyte accumulation in the CNS in the absence of IFN-I signaling.

**Fig. 2:**
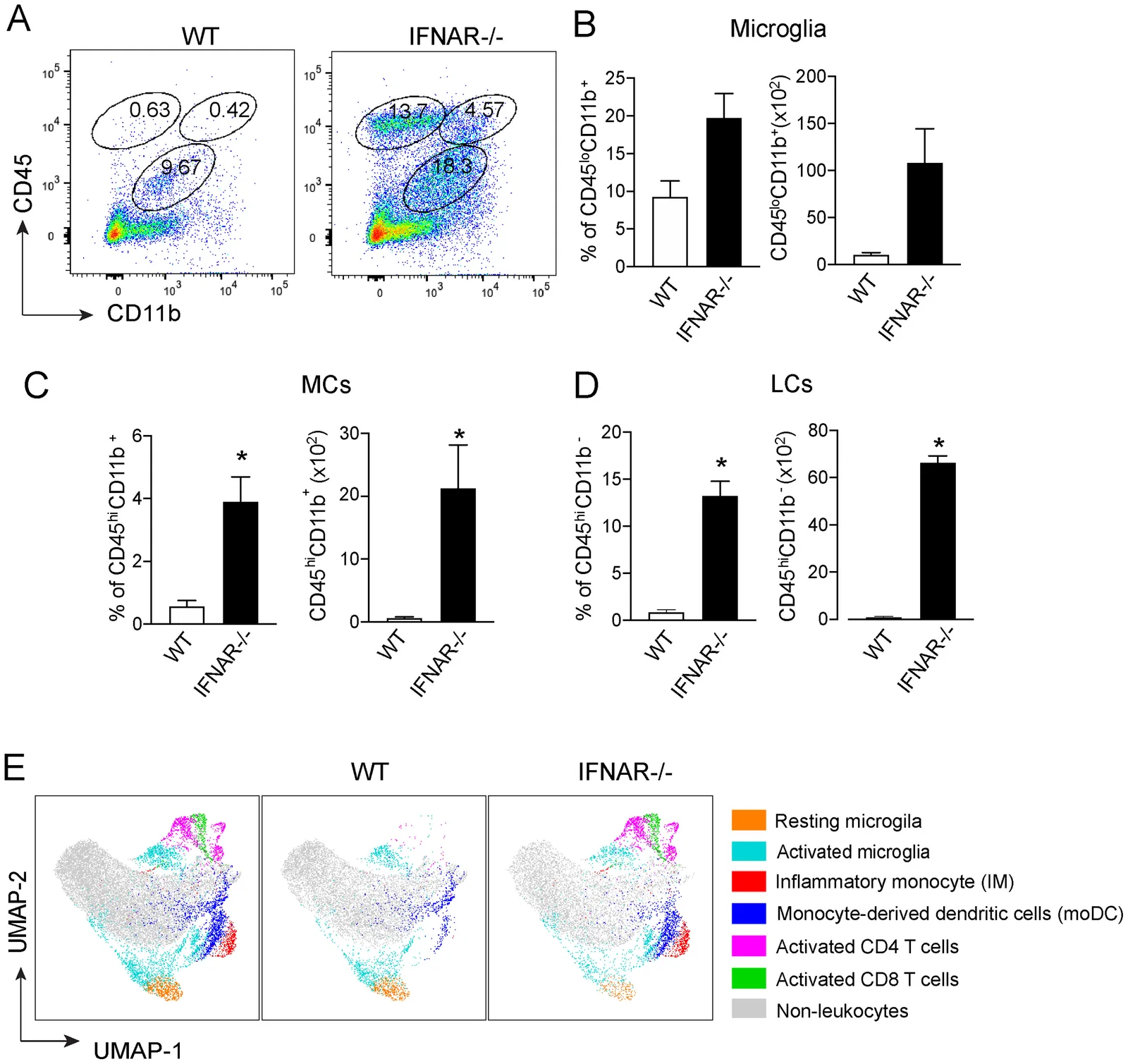
**CNS immune cell populations following ZIKV infection in WT and IFNAR**^−^**^/^**^−^**mice**. At 5 dpi, CNS tissues were harvested from WT and IFNAR^−/−^ mice infected with 1 × 10^5^ PFU of ZIKV. Leukocytes were isolated and stained for flow cytometric analysis. (A) Representative flow-cytometry gating strategy used to identify CNS immune cell populations. Following identification of viable CNS-derived cells, CD45 expression was used to distinguish CD45^lo^ from CD45^hi^ cells. CD45^lo^CD11b^+^ cells were identified as microglia, whereas CD45^hi^CD11b^+^ and CD45^hi^CD11b^−^ cells were identified as myeloid cells (MCs) and lymphoid cells (LCs), respectively. (B–D) Frequency and absolute numbers of microglia (B), MCs (C), and LCs (D), respectively. (E) UMAP plot of CNS-infiltrating immune cells showing seven distinct clusters identified using the X-shift and Cluster Explorer plugins based on the expression of CD45, CD11b, CD11c, Ly6C, MHC II, CD3, CD4, CD8, and CD44. Data are presented as mean ± SEM from two independent experiments (n = 5 mice/group). Statistical comparisons between WT and IFNAR^−/−^ mice were performed using an unpaired Student’s *t*-test. Exact *p*-values were as follows: microglia, frequency *p* = 0.0519 and absolute number *p* = 0.0537; MCs, frequency *p* = 0.0147 and absolute number *p* = 0.0400; LCs, frequency *p* = 0.0013 and absolute number *p* < 0.0001.

Unsupervised UMAP analysis of CNS immune cells revealed seven distinct clusters in IFNAR^−/−^ mice, including: resting microglia (CD45^lo^CD11b^+^CD11c^−^LY6C^−^MHCII^−^), activated microglia (CD45^int^CD11b^+^CD11c^−^LY6C^−^MHCII^+^), inflammatory monocyte (CD45^hi^CD11b^+^LY6C^hi^MHCII^+^), monocyte-derived dendritic cells (moDCs) (CD45^hi^CD11b^+^CD11c^+^LY6C^lo^MHCII^+^), activated CD4^+^ T cells (CD45^hi^CD3^+^CD4^+^CD44^hi^), activated CD8^+^ T cells (CD45^hi^CD3^+^CD8^+^CD44^hi^), and non-leukocyte cells (CD45^−^). In contrast, only the four clusters - resting and activated microglia, moDCs, and CD45^−^ non-leuckocyte cells - were detected in the CNS of infected WT mice (Fig. 2E).

At earlier time points (48 and 72 hpi), IFNAR^−/−^ mice already showed a marked increased infiltration of CD45^hi^CD11b^+^ MCs into the CNS compared to WT controls, beginning as early as 48 hpi, and persisting through 72 hpi (Figs. 3 A and B). In contrast, the absolute number CD45^lo^CD11b^+^ microglia and CD11b^−^CD45^hi^ LCs were comparable between groups across the time points analyzed (Fig. 3 A and B).

**Fig. 3:**
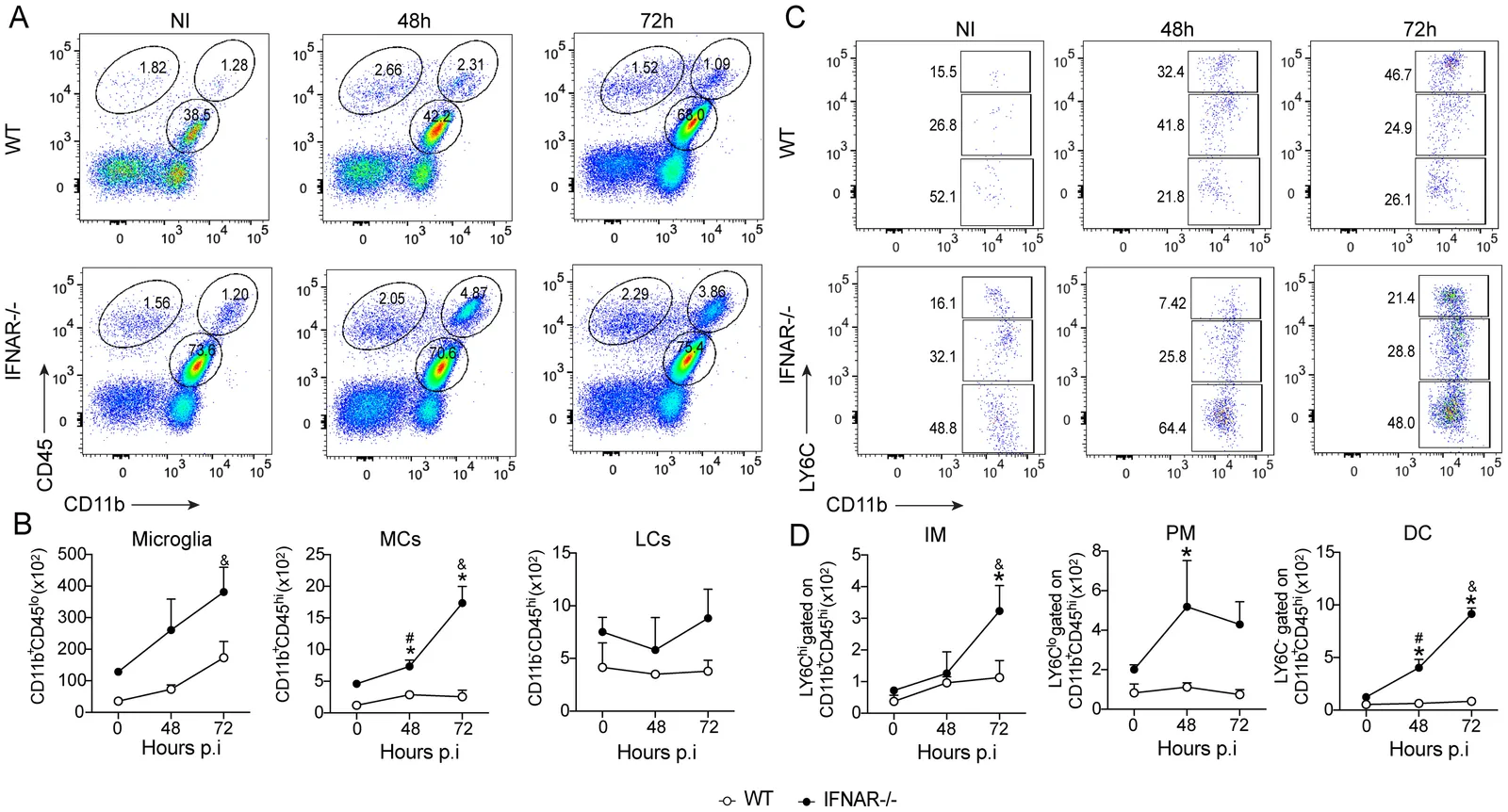
**Lack of IFN-I signaling enhances inflammatory monocyte migration to the CNS during ZIKV infection**. WT and IFNAR^−/−^ mice were infected with 1 × 10^5^ PFU of ZIKV, and CNS samples were harvested at 48 and 72 hpi for leukocyte characterization. (A) Representative dot plots showing CD11b and CD45 expression. (B) Absolute number of CD45^lo^CD11b^+^ microglia, CD45^hi^CD11b^+^ myeloid cells (MCs), and CD45^hi^ CD11b^−^ lymphoid cells (LCs) over time. (C) Representative dot plots showing LY6C expression gated on MCs (CD45^hi^CD11b^+^), and quantification of absolute numbers of inflammatory monocytes (IM; Ly6C^hi^), patrolling monocytes (PM; LyC6^lo^), and dendritic cells (DCs, Ly6C^−^) subsets. Experiments were performed twice, with four mice per group, and the data are presented as mean ± SEM. Statistical significance (#, &,*P < 0.05) is indicated.

Further characterization of the MCs compartment showed that it was composed of inflammatory monocytes (IM; Ly6C^hi^), patrolling monocytes (PM; LyC6^lo^), and dendritic cells (DCs, Ly6C^−^) (Figs. 3C and D). Notably, IFNAR^−/−^ mice exhibited increased recruitment of inflammatory monocyte (MI) at 72 hpi) compared to WT control mice (Fig. 3 C and D. Patrolling monocytes (PM) were already significantly elevated at 48 hpi in IFNAR^−/−^ mice compared to WT mice. Likewise, DCs were elevated in the CNS beginning at 48 hpi and continued to accumulate through 72 hpi in the absence of IFN-I signaling. These DCs (LY6C^−^) also expressed high levels CD11c and MHC class II, consistent with an activated phenotype (Supplementary Figure 1 A and B). These findings indicate that impaired IFN-I signaling is associated with increased CNS viral burden and enhanced accumulation of inflammatory leukocytes during ZIKV infection, which may contribute to CNS immunopathology.

### 3.3 IFNAR deficiency exacerbates pro-inflammatory responses in the CNS during ZIKV infection

As the lack of IFN-I signaling led to heightened neuroinflammation during ZIKV infection, we assessed the expression and production of inflammatory mediators in the CNS. We found that transcripts of Ccl2, Ccl4, and Ccl5, as well as protein levels of CCL2, CCL4, and CCL5, were significantly increased in the CNS of IFNAR^−/−^ mice compared to WT controls, as determined by qPCR and ELISA (Figs. 4A and C). At the protein level, ELISA quantification also revealed elevated levels of pro-inflammatory cytokines, IL-6, IL-1β, TNF, and IFN-γ in the CNS of IFNAR^−/−^ mice (Figs. 4B and D). In addition, increased expression of the inflammatory mediator inducible nitric oxide synthase enzyme (iNOS/NOS-2) was observed in CNS tissue of IFNAR^−/−^ mice compared to WT controls (Figs. 4E). Immunohistochemistry further confirmed increased iNOS expression in the CNS of IFNAR^−/−^-infected mice (Fig. 4 F). Collectively, these data indicate that impaired IFN-I signaling is associated with an enhanced inflammatory milieu in the CNS during ZIKV infection, characterized by increased expression of pro-inflammatory cytokines, chemokines, and iNOS, which may contribute to CNS immunopathology in IFNAR-deficient mice.

**Fig. 4:**
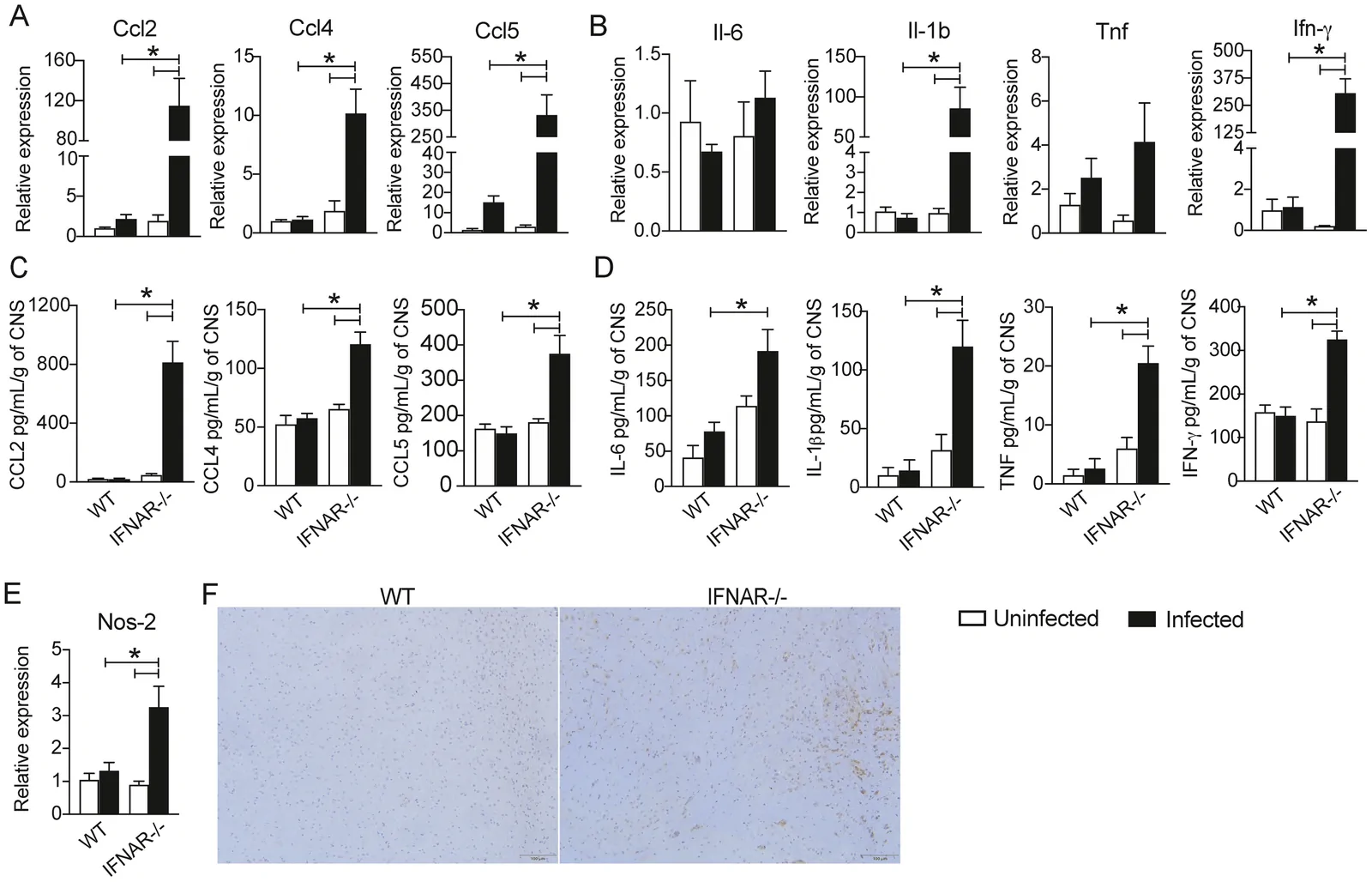
**Impaired IFN-I signaling increases chemokine and cytokine levels in the CNS during ZIKV infection**. WT and IFNAR^−/^- mice were infected with 1 × 10^5^ PFU of ZIKV and 5 days later, the brains were harvested and the mRNA isolated. The mRNA expression of the chemokines CCL2, CCL4, and CCL5 (A), and of the pro-inflammatory cytokines IL-6, IL-1β, TNF, and IFN-γ cytokines (B) was assessed by qPCR. Gene expression was normalized to HPRT using the 2^−ΔΔCt method. Protein levels of the same cytokines and chemokines were measured by ELISA in CNS homogenates (C and D). NOS-2 mRNA expression in CNS tissue was assessed by qPCR (E). (F) Representative immunohistochemistry images showing NOS-2 expression are showed (scale bars, 100 μm). Data are represented mean ± SEM (n = 5/group). Experiments were repeated twice. *P < 0.05.

### 3.4 ZIKV infection is associated distinct activation and cell death patterns in CNS-resident and infiltrating immune cells

An excessive inflammatory response in the CNS can be detrimental to the host (Komarasamy et al., 2022). Consistent with this, ZIKV-infected IFNAR^−/−^ mice exhibited widespread detection of cleaved caspase-3 throughout the CNS, indicative of increased apoptosis, whereas WT mice showed no detectable caspase-3 staining (Fig. 5A). Flow cytometry analysis of the leukocytes from CNS revealed that CD45^lo^CD11b^+^ microglia displayed a significantly higher proportion of dead cells, as determined by Live/Dead staining. In contrast, other subsets, including CD45^hi^CD11b^+^ MCs and CD45^hi^CD11b^−^ LCs, remained predominantly viable (Live/Dead negative) (Fig. 5B).

**Fig. 5:**
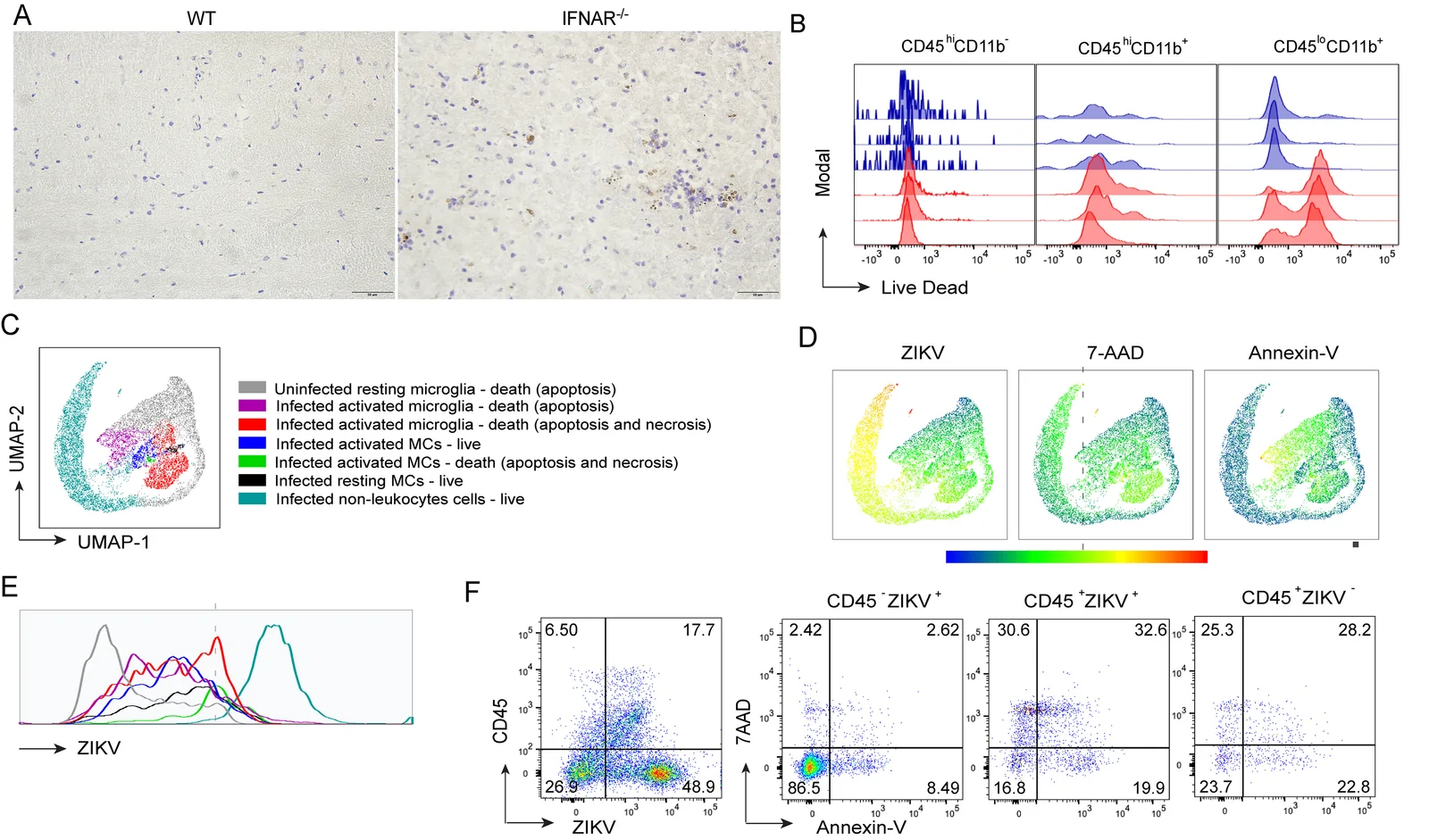
**IFN-I signaling prevents immune cell death in the CNS during ZIKV infection**. WT and IFNAR^−/−^ mice were infected intraperitoneally with 1 × 10^5^ PFU of ZIKV, and brains were harvested at 5 dpi for immunohistochemical analysis and flow cytometry. (A) Representative images showing caspase-3 expression in CNS tissue from WT and IFNAR^−/−^ mice (scale bars = 50 μm). (B) Representative histograms showing Live/Dead staining of CD45^hi^CD11b^−^, CD45^hi^CD11b^+^, and CD45^lo^CD11b^+^ CNS cell populations. (C) UMAP plot of CNS-isolated cells from IFNAR^−/−^ mice showing seven distinct clusters identified based on the expression of CD45, CD11b, MHC II, 7-AAD, Annexin V, and ZIKV antigen using FlowSOM and Cluster Explorer plugins. (D–E) UMAP plots and representative histograms showing the expression of ZIKV antigen, 7-AAD, and Annexin V across the identified cell clusters. (F) Representative dot plots showing ZIKV antigen-positive and antigen-negative CD45^+^ and CD45^−^ cells, as well as 7-AAD and Annexin V expression across different leukocyte and non-leukocyte populations. Data are representative of two independent experiments, with three to five mice per group.

We next investigated which CNS cell populations were ZIKV antigen-positive and assessed their viability. Flow cytometry analysis revealed that activated and ZIKV antigen-positive microglia (CD45^lo^CD11b^+^MHCII^+^ZIKV^+^) exhibited both apoptotic (Annexin V^+^) and late apoptotic/necrotic (Annexin V^+^7AA-D^+^) or (7-AAD^+^) cell-death phenotypes (Fig. 5C-F). Notably, resting and ZIKV antigen-negative microglia (CD45^lo^CD11b^+^MHCII^−^ZIKV^−^) also displayed increased apoptosis (Fig. 5C). This finding is consistent with the possibility that inflammatory signals generated during ZIKV infection may contribute to apoptosis in uninfected microglia, although the present data do not establish a specific bystander mechanism.

Among infiltrating MCs, two major subpopulations were identified: activated/ZIKV antigen-positive (CD45^int^CD11b^+^LY6C^+^MHCII^+^ZIKV^+^) and non-activated/ZIKV antigen-positive (CD45^int^CD11b^+^LY6C^+^MHCII^−^ZIKV^+^) cells (Fig. 5 C). The activated, ZIKV antigen-positive MCs included both viable cells and cells exhibiting apoptotic or necrotic, whereas he non-activated yet ZIKV antigen-positive population remained predominantly viable (Fig. 5C).

Interestingly, CD45^−^ non-leukocyte CNS cells were also highly positive for ZIKV antigen but largely retained viability and represented the main ZIKV antigen-positive population within the CNS (Figs. 5 C, D and E). In contrast, microglia, including both ZIKV antigen-positive and ZIKV antigen-negative cells, were particularly susceptible to apoptosis and necrosis (Figs. 5 D and F). Although most infiltrating MCs were ZIKV antigen-positive, only a small fraction exhibited cell-death phenotypes (Figs. 5 C, D and F).

Together, these findings indicate that ZIKV infection is associated with distinct patterns of activation and cell death among CNS-resident and infiltrating immune cells. Microglia are particularly susceptible to cell death, whereas infiltrating myeloid cells displayed heterogeneous outcomes depending on their activation status. The high frequency of ZIKV antigen-positive cells within the viable CD45^−^ compartment indicates that this compartment accounted for a major proportion of ZIKV antigen-positive cells detected in the CNS during the acute phase of infection. However, the present findings do not establish viral persistence or identify the specific cell types comprising this CD45^−^ population.

## 4 Discussion

Our findings confirm the well-established importance of type I interferon (IFN-I) signaling in limiting ZIKV replication and disease severity. Previous studies using IFNAR-deficient mice have demonstrated increased viral replication, severe neurological disease, and mortality following ZIKV infection, consistent with the established antiviral role of IFN-I signaling (Lazear et al., 2016; Jurado et al., 2018; Rossi et al., 2016). Thus, these aspects of our findings should be considered confirmatory rather than novel. In agreement with these previous observations, IFNAR-deficient mice in our study exhibited increased viral burden, profound weight loss, elevated clinical scores, and lethality, whereas WT animals remained largely asymptomatic. These findings reinforce the critical antiviral function of IFN-I signaling in controlling ZIKV infection and limiting disease severity.

Importantly, we do not interpret the increased susceptibility of IFNAR-deficient mice to ZIKV infection as a novel finding. Rather, this established phenotype provided the experimental framework to investigate how impaired IFN-I signaling is associated with the magnitude and composition of the CNS inflammatory response. In this context, our study extends previous observations by characterizing the cellular landscape of CNS inflammation, including the accumulation and phenotypic profile of infiltrating myeloid and lymphoid populations, as well as their association with ZIKV antigen detection and cell death.

We observed early and robust upregulation of IFN-α and IFN-β in the CNS following ZIKV infection, consistent with previous evidence of IFN-I antiviral activity during flavivirus infections (Samuel and Diamond, 2005; Lubick et al., 2015). In mouse models, IFN-I restricts early replication and dissemination of West Nile virus (WNV) (Samuel and Diamond, 2005), whereas flaviviruses, including ZIKV, have evolved mechanisms to antagonize IFN-I responses and interfere with IFNAR-mediated signaling (Lubick et al., 2015). These observations reinforce the central role of IFN-I signaling in restricting flavivirus infection and provide context for the severe phenotype observed in IFNAR-deficient animals.

Beyond this established antiviral function, our data provide additional insight into the relationship between IFN-I signaling and the cellular inflammatory response in the CNS. Although the increased viral burden observed in IFNAR-deficient mice is consistent with the established antiviral role of IFN-I, the marked accumulation of inflammatory cells in the CNS highlights an additional immunoregulatory dimension of this pathway during ZIKV infection. Using flow cytometry and high-dimensional analysis, we identified diverse immune populations, including inflammatory monocytes, monocyte-derived dendritic cells (moDCs), and activated CD4⁺ and CD8⁺ T cells. Infiltration of innate immune cells was detectable as early as 48 hours post-infection and persisted through 72 hours, indicating a pronounced inflammatory response in the absence of IFNAR signaling. Previous studies have also demonstrated substantial immune-cell infiltration in the CNS following ZIKV infection, including both innate and adaptive immune populations (Jurado et al., 2018; Hayashida et al., 2019). Our findings extend these observations by providing a more detailed characterization of the timing, magnitude, and cellular composition of CNS leukocyte accumulation in the context of impaired IFN-I signaling. In particular, the identification of distinct inflammatory monocyte, dendritic-cell, and activated T-cell populations provides complementary information to previous studies of ZIKV-associated CNS inflammation.

The increased accumulation of inflammatory cells in the absence of IFNAR signaling was associated with a pronounced neuroinflammatory phenotype. However, these findings should be interpreted in the context of the substantially higher viral burden observed in IFNAR-deficient mice. Although impaired IFN-I signaling was associated with enhanced accumulation of inflammatory myeloid and lymphoid cells in the CNS, the present experimental design does not allow us to determine whether IFN-I signaling directly regulates leukocyte recruitment independently of viral replication. The increased leukocyte accumulation may therefore be driven, at least in part, by the higher viral burden and the associated inflammatory signals in the CNS. Rather, our findings support a model in which impaired IFN-I signaling permits increased viral replication and is associated with an altered inflammatory environment, resulting in enhanced accumulation of immune cells in the CNS and contributing to disease severity. Thus, while our data demonstrate an association between impaired IFN-I signaling, increased viral burden, and enhanced CNS inflammation, they do not establish a direct causal effect of IFN-I on leukocyte recruitment. This interpretation is consistent with previous studies demonstrating that both viral replication and immune responses can contribute to ZIKV-associated neuropathology (Jurado et al., 2018; Hayashida et al., 2019).

In addition to increased immune-cell infiltration, IFNAR-deficient mice exhibited elevated levels of chemokines, including CCL2, CCL4, and CCL5, and proinflammatory cytokines, including IL-6, IL-1β, TNF, and IFN-γ, together with increased expression of inducible nitric oxide synthase (iNOS) in the CNS. These mediators are well-established regulators of immune-cell recruitment and activation during neuroinflammation and may contribute to the enhanced inflammatory environment observed in IFNAR-deficient mice. Given that IFN-γ is an established inducer of iNOS during CNS inflammation (Sonar and Lal, 2019), increased iNOS expression may contribute to the inflammatory environment and CNS pathology observed in these animals.

IL-6 has also been implicated in the pathogenesis of viral encephalitis, with elevated levels associated with neuronal injury and adverse disease outcomes in JEV models (Winter et al., 2004; Ghoshal et al., 2007). Similarly, excessive production of nitric oxide (NO) through iNOS has been linked to CNS damage. Although NO has important physiological functions, excessive production during inflammation can promote neurotoxicity, particularly through the generation of peroxynitrite and other reactive nitrogen species that can impair mitochondrial function and damage cellular membranes (Bogdan, 2001; Calabrese et al., 2007). In experimental models of WNV and tick-borne encephalitis virus, increased nitric oxide activity has been associated with greater disease severity and neuronal damage (Akaike and Maeda, 2000; Getts et al., 2012).

Collectively, these data indicate that impaired IFN-I signaling is associated with an enhanced inflammatory milieu in the CNS during ZIKV infection, characterized by increased expression of pro-inflammatory cytokines, chemokines, and iNOS. Although the cellular sources of these inflammatory mediators have been well characterized in the literature, our analyses were performed using whole-brain tissue and therefore do not allow us to determine the relative contribution of individual CNS-resident or infiltrating cell populations to the increased mediator levels observed in IFNAR^−/−^ mice. In addition, our data do not establish whether the production of these mediators is directly associated with ZIKV antigen-positive cells or occurs indirectly through inflammatory interactions among infected and uninfected cells.

Consistent with these inflammatory changes, we observed pronounced activation of cleaved caspase-3 and parenchymal apoptosis in the CNS of IFNAR-deficient mice, particularly affecting microglia. This finding is consistent with reports of increased apoptosis in congenital ZIKV-infected human brain tissue (Azevedo et al., 2018). A particularly striking finding of this study was the differential susceptibility of CNS-resident and infiltrating immune cells to ZIKV-associated cell death. While microglia, both infected and uninfected, underwent substantial apoptosis and necrosis, infiltrating myeloid cells remained largely viable despite being infected.

Previous studies have shown that microglia are susceptible to virus-induced cell death and that their depletion exacerbates ZIKV-induced defects in neurogenesis and enhances viral replication in the brain (Dang et al., 2016; Retallack et al., 2016; Li et al., 2016). In other neurotropic viral infections, inflammatory signaling pathways have also been implicated in microglial apoptosis. For example, TLR2 signaling has been associated with microglial apoptosis during HSV-1 infection (Aravalli et al., 2007), while activation of the cGAS/STING pathway has been linked to inflammatory responses and cell death in the context of viral encephalitis (Reinert et al., 2021). Similarly, SARS-CoV-2 infection has been associated with pro-inflammatory activation and apoptosis of microglial cells (Jeong et al., 2022).

In contrast, infiltrating myeloid cells, including monocytes, monocyte-derived macrophages (MDMs), and dendritic cells, displayed notable resistance to cell death during ZIKV infection. Human monocytes and MDMs can sustain viral replication without detectable cytopathic effects, as indicated by stable markers of necrosis comparable to those observed in uninfected controls (Pagani et al., 2025). When polarized toward a pro-inflammatory M1 phenotype, these MDMs further restrict ZIKV replication while maintaining viability, highlighting a potential functional resistance to infection-associated cytotoxicity (Pagani et al., 2025).

Interestingly, apoptosis was also detected among ZIKV-negative microglia, raising the possibility that cell death may occur through mechanisms that are not directly dependent on viral infection. This finding is consistent with the possibility that inflammatory signals generated during ZIKV infection may contribute to apoptosis in uninfected microglia. However, because we did not directly dissect virus-dependent cytotoxicity from cytokine- or inflammation-mediated injury, the contribution of inflammatory signals to apoptosis in ZIKV-negative microglia remains speculative. Further mechanistic studies will be required to determine whether inflammatory mediators contribute to apoptosis in ZIKV-negative microglia during ZIKV infection. Together, these findings highlight a differential susceptibility of CNS immune populations to infection-associated cell death and suggest that cellular context may influence the outcome of ZIKV infection.

Interestingly, most ZIKV-positive cells detected in the CNS were non-leukocyte (CD45⁻) populations and exhibited high viability. This observation indicates that cells within the CD45⁻ compartment can contain viral antigen while maintaining viability during the acute phase of infection. However, CD45⁻ is a broad phenotypic designation that encompasses multiple non-hematopoietic cell populations and therefore does not allow identification of the specific CNS cell types containing viral antigen. Because the present study focused on the acute phase of infection and did not include long-term follow-up, the detection of viable ZIKV antigen-positive CD45⁻ cells should not be interpreted as evidence of a viral reservoir or viral persistence. Rather, these findings demonstrate the presence of viable ZIKV antigen-positive cells within the CD45⁻ compartment during acute infection. Future studies incorporating lineage-specific markers and longitudinal analyses will be necessary to determine the identity of these cells and whether any of these populations can support or contribute to viral persistence.

An important limitation of this study is the use of a global IFNAR-deficient model, which does not allow us to distinguish the direct contribution of impaired IFN-I signaling in CNS-resident cells from indirect effects resulting from altered IFN-I responses in peripheral immune cells. Type I interferon signaling in both CNS-resident and peripheral cells can influence the outcome of neurotropic viral infections and the associated inflammatory response (Daniels et al., 2017; Lazear et al., 2016). Therefore, the marked accumulation of leukocytes observed in the CNS of IFNAR-deficient mice may reflect combined effects of altered peripheral immune responses and impaired IFN-I signaling within CNS-resident cells.

In addition, blood-brain barrier (BBB) permeability was not directly assessed in the present study. Thus, we cannot determine whether alterations in BBB integrity contributed to the enhanced leukocyte infiltration observed in IFNAR-deficient animals. Previous studies have demonstrated that IFN-I signaling and BBB integrity can influence immune-cell entry into the CNS during neuroinflammatory and viral infections (Daniels et al., 2017). Therefore, altered BBB function represents a potential mechanism that may contribute to the enhanced leukocyte accumulation observed in our model, although this possibility cannot be directly established from the present data. Future studies using cell-specific IFNAR-deficient models, together with direct assessment of BBB permeability, will be important to determine the relative contributions of CNS-resident and peripheral IFN-I signaling, as well as the potential role of BBB dysfunction in the enhanced CNS inflammatory response.

The relatively high ZIKV inoculum used in this study was selected because C57BL/6 mice are relatively resistant to ZIKV infection and may exhibit limited viral replication and disease following infection (Lazear et al., 2016). A higher viral dose was therefore selected to establish a sufficiently robust infection and induce detectable pathological changes, enabling the evaluation of CNS inflammation and disease progression. Nevertheless, the relatively high inoculum administered intraperitoneally does not reproduce the conditions of natural mosquito-borne transmission. This represents an important limitation when extrapolating the viral kinetics, viral burden, inflammatory response, and disease severity observed in this experimental model to natural ZIKV infection.

Although the present study focused on the CNS, our findings should be considered within the broader context of ZIKV systemic pathogenesis. Recent studies have shown that ZIKV can infect and induce pathological alterations in tissues beyond the CNS, including reproductive, ocular, cardiovascular, and placental tissues (Giraldo et al., 2023; Labib and Chigbu, 2022; Scatularo et al., 2022; Rabelo et al., 2020; Liu et al., 2023). These findings highlight the diversity of tissue-specific responses and pathological mechanisms associated with ZIKV infection. Although we did not investigate IFN-I signaling in these tissues, its established role in controlling ZIKV replication suggests that antiviral and inflammatory responses may contribute to disease manifestations across different organ systems (Giraldo et al., 2023). Further studies are needed to determine how impaired IFN-I signaling influences ZIKV pathogenesis beyond the CNS.

Overall, our findings reinforce the established antiviral role of IFN-I signaling in controlling ZIKV replication and disease severity while providing additional insight into its association with the cellular inflammatory response in the CNS. Impaired IFN-I signaling was associated with increased viral burden, marked accumulation of inflammatory leukocytes, enhanced expression of inflammatory mediators, and increased cell death. The differential susceptibility of microglia and infiltrating myeloid cells to cell death, together with the detection of viable ZIKV antigen-positive cells within the CD45⁻ compartment, further highlights the complexity of the cellular response to ZIKV infection in the CNS.

Taken together, this study extends previous work on the antiviral function of IFN-I by characterizing the temporal and cellular features of CNS inflammation associated with impaired IFN-I signaling during ZIKV infection. While the increased susceptibility of IFNAR-deficient mice to ZIKV infection and severe disease is consistent with previous reports (Lazear et al., 2016; Rossi et al., 2016), our findings provide complementary information on the cellular composition of the CNS inflammatory response and the differential association of viral antigen detection, cellular activation, and cell death among CNS immune populations. These findings provide an integrated view of the relationship between viral replication, immune-cell accumulation, inflammatory responses, and cellular injury during ZIKV-associated CNS disease.

## Ethics declaration

This study was conducted in accordance with the ARRIVE (Animal Research: Reporting of In Vivo Experiments) guidelines. This study was approved by the Ethics Committee at the University of São Paulo, Brazil. (Approval No protocol number 152/2017)

## CRediT authorship contribution statement

**Luciana Benevides:** Writing – review & editing, Writing – original draft, Methodology, Investigation, Formal analysis, Data curation, Conceptualization. **Herculano da Silva:** Methodology. **Franciele Pioto:** Methodology. **Thais da Silva Rigoni:** Methodology. **Mariela Pires Cabral Piccin:** Methodology. **Juliano de Paula Souza:** Methodology. **Eurico Arruda:** Methodology, Conceptualization. **Vanessa Carregaro:** Writing – original draft, Methodology, Conceptualization. **João S. Silva:** Writing – review & editing, Writing – original draft, Supervision, Resources, Project administration, Investigation, Funding acquisition, Conceptualization.

## Declaration of competing interest

The authors declare that they have no known competing financial interests or personal relationships that could have appeared to influence the work reported in this paper.

## Data Availability

No data was used for the research described in the article.
