# Supplementary material for: Type I interferon mitigates ZIKV-induced neuroinflammation and cell death in the central nervous system
Source: Virus Res. 2026 Oct 4;373:199814. doi: 10.1016/j.virusres.2026.199814 (PMC13679374; doi:10.1016/j.virusres.2026.199814)
Supplement: Supplementary file 1 [file mmc1.docx]

Supplementary material

Supplementary Table 1. Murine primer sequences used in the study

| **Gene** | **Foward (5’-3”)** | **Reverse (5”-3’)** |
| --- | --- | --- |
| IFNa | ACCTCCACCAGCGCAGCTCA | CCCCACCTGCTGCATCAG |
| IFNb | CCACTTGAAGAGCTATTACTGGAGG | TCCAGGCGTAGCTGTTGTACTT |
| CCL2 | CATCCACGTGTTGGCTCA | AACTACAGCTTCTTTGGGACA |
| CCL4 | GTCTCATAGTAATCCATCACAAAGC | CTCTCTCTCCTCTTGCTCGT |
| CCL5 | GCTTTGCCTACCTCTCCCTC | AACACGACTGCAAGATTGGA |
| IL-6 | AAGAAAGACAAAGCCAGAGTCCT | TTTTACCTCTTGGTTGAAGATATGAA |
| IL-1b | CTCTTGTTGATGTGCTGCTG | GACCTGTTCTTTGAAGTTGACG |
| TNF | AGGGATGAAGTTCCCAAATG | GGCTTGTCACTCGAATTTTGAGA |
| IFN-g | CATGGCTGTTTCTGGCTGTTAC | CCAGTTCCTCCAGATATCCAAGA |
| Nos-2 | CGAAACGCTTCACTTCCAA | TCAGCCTATATTGCTGTGGCT |
| Hprt | AACAAAGTCTGGCCTGTATCC | CCCCAAAATGGTTAAGGTTGC |

**Supplementary Figure 1. Expression of CD11c and MHCII in dendritic cells infiltrating the CNS during ZIKV infection. At 5 dpi, CNS tissues were harvested from** WT and IFNAR^-/-^ mice infected with 1x10^5^ PFU of ZIKV. Leukocytes were isolated and stained for flow cytometry analysis. Dot plot represent (A) of percentage and (B) the absolute numbers of CD11c^+^MHCII^+^ cells gated on CD45^hi^CD11b^+^Ly6C^-^population. Data are presented as mean ± SEM from two independent experiments (n = 5/group). *P < 0.05.
